# Irradiation free conditioning regimen is associated with high relapse rate in Egyptian patients with acute lymphoblastic leukemia following allogeneic hematopoietic stem cell transplantation

**DOI:** 10.1186/s43046-020-00042-4

**Published:** 2020-06-15

**Authors:** Mona Mahrous Abdelaty, Amr Gawaly, Gamal M. Fathy, Ibrahim Kabbash, Atef Taha

**Affiliations:** 1 Internal Medicine Department, Hematology/Bone marrow transplantation unit, Tanta University, Tanta, Algharbia Egypt; 2 Nasser Institute Hospital for Research and Treatment, Cairo, Egypt; 3 Public Health and Community Medicine Department, Tanta University, Tanta, Egypt

**Keywords:** Allogeneic hematopoietic stem cell transplantation, Acute lymphoblastic leukemia, Conditioning, Total body irradiation, Busulfan

## Abstract

**Background:**

Allogeneic hematopoietic stem cell transplantation (Allo-HSCT) is a curative treatment for adult patients with acute lymphoblastic leukemia (ALL). Cyclophosphamide plus total body irradiation (TBI/Cy) or plus busulfan (Bu/Cy) is a widely used pre-transplant conditioning regimen for ALL. We retrospectively compared the overall survival (OS), disease-free survival (DFS), and other transplant outcomes of allo-HSCT in 119 adult patients with ALL who received an HLA-matched sibling allo-HSCT using TBI-based versus non-TBI-based conditioning regimens. Patients were divided into two groups by their conditioning regimen: TBI/Cy or Bu/Cy.

**Results:**

Median OS was 11 months in the TBI/Cy group and 6.2 months in the Bu/Cy group. Median DFS was 11.1 months in the TBI group versus 6.8 months in the Bu group, without a statistically significant difference. A higher risk of relapse was observed with the Bu/Cy regimen (HR 2.709, CI 95% 1.106 to 6.638, *p* = 0.029). Patients who received a transplant in ≥ CR2 were associated with poor DFS.

**Conclusion:**

Despite the high relapse rate in the non-TBI regimen (Bu/Cy), both regimens had no statistically significant differences in OS, DFS, and NRM. Additional prospective studies are indeed warranted to evaluate the long-term outcomes of radiation-free regimens, including oral and intravenous busulfan, and compare these regimens with TBI-based ones.

## Background

ALL generally has an excellent prognosis in children with promising chemotherapy regimens [1]. In contrast, it is still challenging in adults as it is associated with poor survival outcomes and high relapse rates when treated with chemotherapy alone. Allo-HSCT is generally considered a lifesaving option that is able to consolidate remission resulting in long-term DFS is adults [2].

Combinations of cyclophosphamide with either TBI or busulfan are the most commonly used myeloablative conditioning regimens (MAC) for allo-HSCT in adult ALL [3]. TBI has dual immunosuppressive and anti-leukemic properties with the ability to reach the hidden sites. It has expected better survival outcomes without an increase in relapse or transplant-related mortality (TRM). Unfortunately, at higher doses, TBI is associated with a potential risk of early and late complications [4].

Although busulfan-containing regimens can produce comparable outcomes with lower TRM, the oral form has wide variability in the absorption and metabolism. Also, high relapse rates have been observed with low levels of busulfan, while many risks including veno-occlusive disease (VOD) have been related to higher levels [5].

Many studies have been carried out to evaluate the effect of different regimens on transplant outcomes, but the ideal conditioning regimen for adult ALL patients remains unknown [3]. This retrospective study aims to compare transplant outcomes in ALL patients using the TBI/Cy and oral Bu/Cy as MAC regimens.

## Methods

A retrospective cohort study was done on data obtained from a tertiary center’s bone marrow transplantation unit registry. The medical files of adult patients with ALL who received a transplant during the period 2000 to 2016 were reviewed. Patients aged 19 years or above at the time of first allo-HSCT from an HLA-matched sibling donor using TBI/Cy or Bu/Cy as a MAC regimen were included, either in first complete remission (CR1), CR2, or beyond. All patients had adequate performance scale and negative serology for the hepatitis B virus and the human immune deficiency virus (HIV).

Patients aged 60 years or above and those who received syngeneic or haploidentical allo-HSCT were excluded.

Detailed history has been reviewed, including data about age, sex, time from diagnosis to transplantation, Philadelphia chromosome, and disease status at transplantation time, associated comorbidities, viral status, donor type, stem cell source, CD34 cell dose, graft versus host disease (GVHD) prophylaxis, and transfusion requirements. Allo-HSCT outcome was assessed with the following parameters: time to engraftment, acute and chronic GVHD, incidence and severity of infections, and conditioning regimen-related toxicities. Survival outcomes included OS, DFS, NRM, and relapse.

### Conditioning regimens

Patients included in our study were divided into two groups based on their conditioning regimens:

Group 1 included ALL patients who received allo HSCT from 2000 to 2015 and received TBI/Cy. The TBI dose was 12 Gy fractionated over 5 days (from day –10 to day –6). Cy was administrated as 30 mg/day (from day –5 to day –2 of the stem cell infusion).

Group 2 included ALL patients who received allo HSCT from 2000 to 2016 and received Bu/Cy. The dose of oral busulfan was 16 mg/kg total dose over 4 days to be given as 4 mg/day orally. The Cy dosage over 4 days was 120 mg/kg IV to be given as 30 mg/day.

### GVHD prophylaxis

All patients received prophylaxis for GVHD using methotrexate (MTX) and cyclosporine (CSA). MTX dose was 15 mg/m^2^ IV given on day +1 then changed to 10 mg/m^2^ on days +3, +6, and +11, as well as folinic acid rescue 15 mg/m^2^ IV TDS for just 24 h on the day after MTX injection. MTX was monitored by the degree of mucositis and bilirubin level with the appropriate drug titration. CSA was administered in two divided doses from day −1 at a dose of 3 mg/kg/day IV that was replaced by an oral form once tolerated. The dose was modified to reach a therapeutic plasma CSA level of 200–250 mg/ml. Renal functions and electrolytes were also monitored with drug titration accordingly.

### Supportive care

Gastric protection by pantoprazole, antiemetics, using ondansetron were initiated at the start of the conditioning regimens and maximized as needed. All patients were given uromitexan guard against cyclophosphamide-induced hemorrhagic cystitis. Seizure prophylaxis by phenytoin was given before and during the administration of busulfan. Local mouth care and prophylaxis against bacterial, fungal, and viral infections were also given to all patients. *Pneumocystis Jirovecii* infection prophylaxis was done by trimethoprim-sulfamethoxazole (stopped in day −2 and re-initiated after engraftment). Preemptive treatment for cytomegalovirus (CMV) reactivation was given according to close molecular monitoring. Supportive irradiated blood products were administrated when needed; whole blood and granulocyte colony-stimulating growth factors (G-CSF) were received in some patients until neutrophil recovery.

### Study endpoints and operational definitions

The primary endpoints were OS and DFS. OS was defined as time to death or last contact for survivors. DFS was identified as a time to treatment failure (relapse or death); for survivors, it was considered as the last contact in remission. The secondary endpoints were relapse and NRM. Relapse was considered as the recurrent appearance of hematological disease. We define NRM as a death in remission.

Engraftment was identified as the first of three consecutive days with an absolute count of neutrophils > 500/μL and platelets > 20,000/μL (unsupported) [6]. The diagnosis and grading of acute and chronic GVHD were based on the established criteria [7, 8]. VOD diagnosis was based on the Baltimore clinical criteria [9]. Mucositis was identified and graded according to the National Cancer Institute (NCI-CTC) criteria [10], and grade 1 was considered as mild, grade 2 as moderate, and grades 3 and 4 as severe mucositis. Renal complications in our study were defined when serum creatinine was ≥ 2mg/dl and/or requiring CSA cessation. CMV infection or reactivation was diagnosed when 2 consecutive titres of CMV DNA are above 500 copies/mL in the presence of GVHD or above 1000 copies/mL in the absence of GVHD.

### Statistical analysis

The collected data were organized, tabulated, and statistically analyzed by SPSS version 24 (Statistical Package for Social Studies) created by IBM, Illinois, Chicago, USA. For numerical values, the range, mean, and standard deviations were calculated. The differences between mean values were tested using (*t*) test while the Mann-Whitney test (*Z*) was used for other variables where data were not normally distributed. For categorical variables, the number and percentage were calculated, and differences between subcategories were tested using the chi-square test. When chi-square was not appropriate, Fisher and Monte Carlo exact tests were used as appropriate. For risk estimation, odds ratio was calculated and its 95% confidence interval. For calculation of the hazard ratio and its 95% confidence interval for the independent effect of each predictor on a certain outcome to occur during the survival period of studied patients, Cox regression was performed. The level of significance was adopted at *p* < 0.05.

## Results

A total of 119 adult patients with ALL received allo-HSCT from HLA-matched sibling donors from January 2000 to December 2016. Seventy-eight patients were transplanted using TBI/Cy, and forty-one patients were transplanted using oral Bu/Cy.

### Baseline characteristics of study population

The baseline characteristics of the seventy-eight patients received TBI/Cy and the forty-one patients received Bu/Cy regimens are described in Table 1. The age ranged from 19–49 to 19–41 years in the TBI/Cy and the Bu/Cy groups, respectively, with a mean age at transplant of 27.64 ± 7.67 in the TBI/Cy and 27.46 ± 7.00 in the Bu/Cy group. In the TBI/Cy group, 56 (71.8%) patients were males, and 22 (28.2%) were females, while in the Bu/Cy group, 73.2% of patients were males. B-ALL was the main subtype in both groups. Most of the patients in both groups had adequate performance. Philadelphia chromosome was positive in 18 (23.1%) patients in the TBI /Cy group and 9 (22%) patients in the Bu/Cy group.

**Table 1:** Baseline characteristics of the study population

Variable	TBI/Cy (*n* = 78)	Bu/Cy (*n* = 41)	*p* value
^*^Age (years)	27.64 ± 7.67 (19–49)	27.46 ± 7.00 (19–41)	0.902
Recipient sex			
Males Females	56 (71.8) 22(28.2)	30 (73.2) 11(26.8)	0.873
Immunophenotype			
B T	52 (66.7) 26 (33.3)	27 (65.9) 14 (34.1)	0.929
Performance status			
0–1 2	75 (96.2) 3 (3.8)	38 (92.7) 3 (7.3)	0.339
Ph chromosome status			
Negative Positive	60 (76.9) 18 (23.1)	32 (78) 9 (22)	0.889
Disease status at transplant			
CR 1 ≥ CR 2	40 (51.3) 38 (48.6)	16 (39) 25 (61)	0.203
Pre-transplant Comorbidities			
No Yes	76 (85.9) 11 (14.1)	37 (90.2) 3 (9.8)	0.497
Donor sex			
Females Males	33 (42.3) 45 (57.7)	22 (53.7) 19 (64.3)	0.238
Donor/recipient CMV seropositivity			
Mismatched Matched	7 (9) 71 (91)	2 (4.9) 39 (95.1)	0.717
^*^Diagnosis to transplant lag period (month)	16.25 (14–137.3)	13.5 (4.9–73.2)	0.114

^*^Values are expressed as mean (range) and *n* (%)

At the time of transplant, 51.3% of patients were in CR1 in the TBI/Cy versus 39% in the Bu/Cy group. Among the 119 patients, only 14 patients were reported to have pre-transplant comorbidities which included diabetes, hypertension, and chronic liver disease. Female donors represented (42.3%) in the TBI group and (53.7%) in the Bu/Cy group. Female donors to male recipients were found in 24 patients in the TBI/Cy group and 16 patients in the Bu/Cy group. Seven patients (9%) in the TBI group had mismatched CMV donor versus 2 patients (4.9%) in the Bu group. The time from diagnosis to transplantation was ranged from 14 to 137.3 months in the TBI group and (4.9–73.2) months in the Bu/Cy group. All these differences were found to be statistically not significant.

### Engraftment and GVHD

One hundred and eleven (93.3%) achieved engraftment ,and only eight patients (6.7%) had primary graft failure. Significant faster engraftment was observed in the Bu/Cy group, a median time to neutrophil engraftment was 17 days (range 9–35) in the TBI /Cy group and 14 days (range10–24) in the Bu/Cy group, while the median time for platelet engraftment was 15 and 14 days in the TBI and the Bu groups, respectively (Table 2). However, no significant difference could be detected between the two groups in the term of engraftment by the multivariate analysis (Table 3). The incidence of acute and GVHD was similar in both groups (Table 2).

**Table 2:** Transplant outcomes

Variables	TBI/Cy (*n* = 78)	Bu/Cy (*n* = 41)	*p* value
Neutrophil engraftment(days)	17(9–35)	14(10–24)	0.002
Platelet engraftment (days)	15(8-47)	14(7–45)	0.017
Acute GVHD			0.723
Negative Positive	52 (66.7) 26 (33.3)	26 (63.4) 15 (36.6)	
Chronic GVHD			0.106
Negative Positive	54 (69.2) 24 (30.8)	34 (82.9) 7 (17.1)	
Relapse after transplant			0.034
No Yes	69 (88.5) 9 (11.5)	30 (73.2) 11 (26.8)	
NRM			0.278
No Yes	48 (61.5) 30 (38.5)	21 (51.2) 20 (48.8)	

Values are expressed as median (range) and *n* (%)

**Table 3:** Multivariate analysis of non-relapse mortality, relapse, and engraftment

Endpoints of the study	HR (95% CI)	***P***
**NRM**		
Variables		
Conditioning	0.692(0.310–1.545)	0.369
Age of recipient	1.019(0.975–1.065)	0.396
Disease status at BMT	1.639(0.775–3.469)	0.196
Ph chromosome status	0.808(0.329–1.984)	0.641
CD34 dose	1.008(0.917–1.108)	0.873
Comorbidities	1.380(0.512–3.721)	0.524
Diagnosis to transplant lag period	1.005(0.989–1.023)	0.531
**Relapse**		
Variables		
Conditioning (TBI/Cy versus Bu/Cy)	2.709(1.106–6.638)	0.029
Age of recipient	0.977(0.914–1.045)	0.500
Philadelphia chromosome status	0.1321(0.437–3.995)	0.622
**Engraftment**		
Variables		
Conditioning (TBI/Cy versus Bu/Cy)	1.229(0.809–1.868)	0.333
Disease status at BMT	1.085(0.774–1.521)	0.635
CD34 dose	0.999(0.942–1.059)	0.970

*HR* hazard ratio, *CI* confidence interval

### Relapse and NRM

Regarding relapse, 16.8% (*n*, 119) of patients developed relapse after transplant. A significantly high relapse rate was observed in the Bu/Cy group (26.8% versus 11.5% in the TBI/ Cy group) (Table 2). The Bu/Cy regimen was identified as an independent risk factor for relapse by the multivariate analysis (Table 3). The incidence of NRM was similar in the two groups, which was proved by the univariate and multivariate analysis (Tables 2 and 3). The most common cause of NRM in the TBI group was sepsis (36.8%), while respiratory failure was the major cause in the Bu group (22.2%).

### DFS and OS

**Fig. 1:**
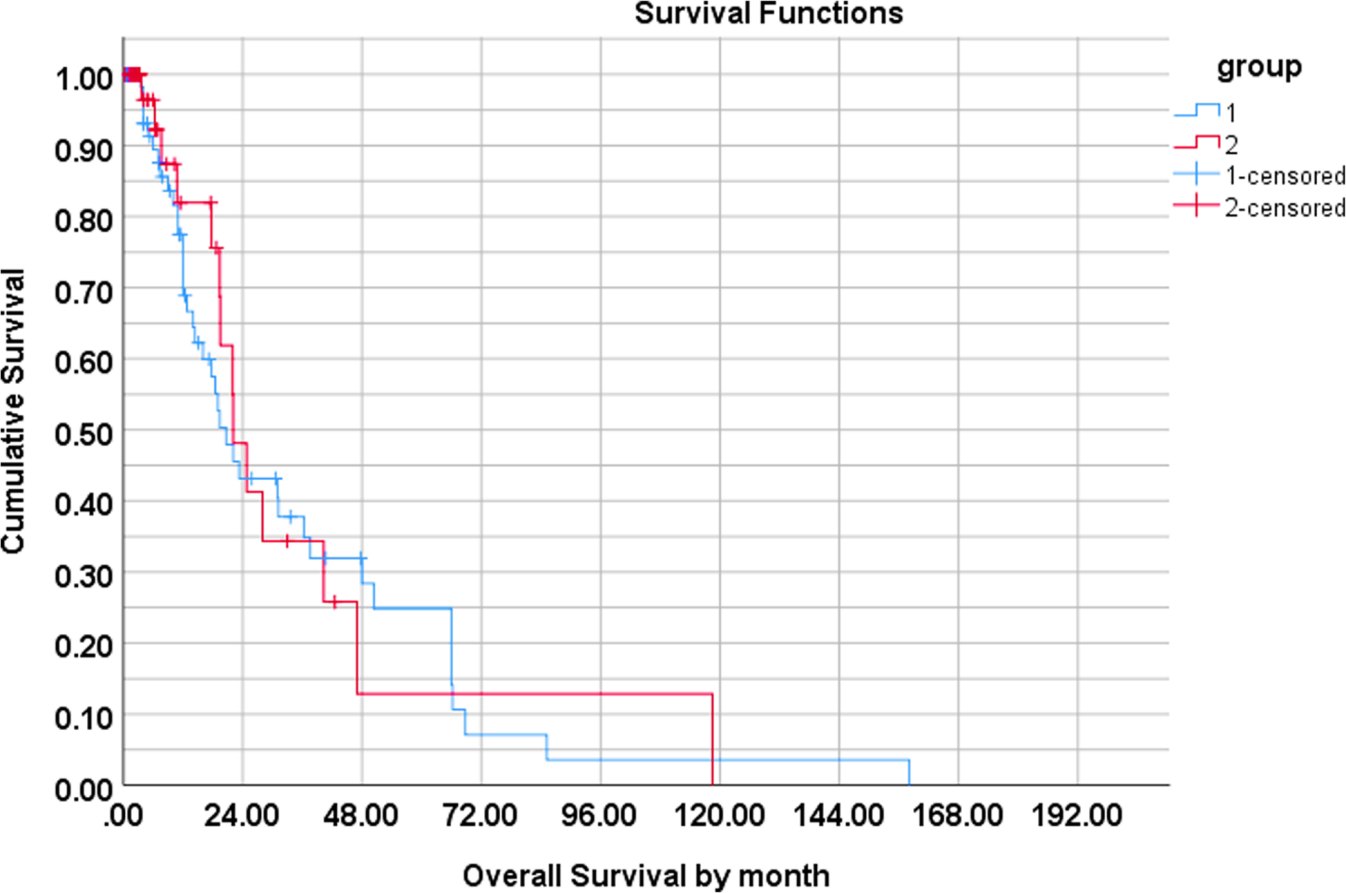
Overall survival by Kaplan-Meier survival curve in ALL patients treated with either TBI/Cy (group 1) or Bu/Cy (group 2)

**Fig. 2:**
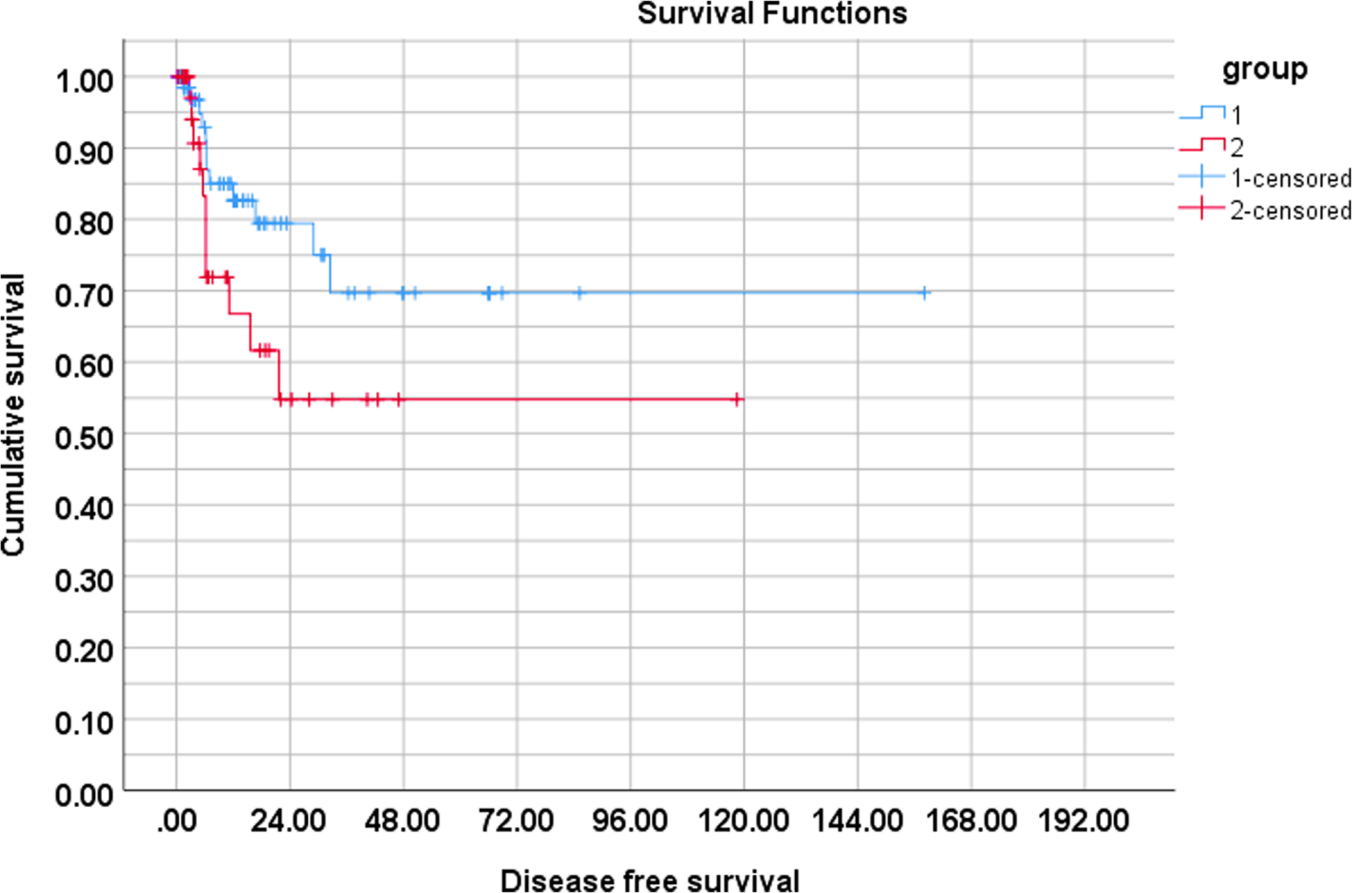
Disease-free survival by Kaplan-Meier survival curve in ALL patients treated with either TBI/Cy (group 1) or Bu/Cy (group 2)

The survival rate was 51.3% and 34.1% in the TBI/Cy and Bu/Cy group, respectively. The follow-up period ranged from (0.3–158) months in the TBI group with a median OS of 11 months, while it ranged from 0.3–118.5 months in the Bu group with a median OS of 6.2 months. The median DFS was 11.1 versus 6.8 months for TBI and Bu groups, respectively. The estimated 2-year OS was 42% in the TBI group and 44% in the Bu group. The estimated 2-year DFS was 80% in the TBI group compared to 55% in the Bu group. All these differences were statistically non-significant (Figs. 1 and 2).

No independent risk factors for low OS were detected by the multivariate analysis. Disease status at transplant was the only independent risk factor for poor DFS in both groups, and patients received a transplant at CR2 or beyond were associated with poor DFS, HR, 3.670 (CI 95% 1.500–8.978 and *p* 0.004). However, the disease status at transplant had no independent effect on relapse risk, OR, 0.420; CI 95% 0.149–1.182 (Tables 4 and 5).

**Table 4:** Multivariate analysis of overall survival and disease-free survival

Endpoints of the study	HR (95% CI)	***P***
**DFS**		
Variables		
Type of conditioning	1.391(0.537–3.600)	0.497
Age of recipient	0.982(0.898–1.073)	0.685
Sex of recipient	0.381(0.115–1.263)	0.115
Disease status at BMT(CR1 vs ≥ CR2)	3.670(1.500–8.978)	0.004
Ph chromosome status	1.303(0.387–4.379)	0.669
Diagnosis to transplant lag	0.961(0.902–1.002)	0.064
**OS**		
Variables		
Conditioning (TBI/Cy vs Bu/cy)	0.764(0.371–1.573)	0.466
Age of recipient (≤30 vs ≥ 30)	1.014(0.974–1.056)	0.489
Sex of recipient (Female vs male)	1.461(0.709–3.013)	0.304
Disease status at BMT(CR1 vs ≥ CR2)	1.660(0.813–3.390)	0.164
Philadelphia chromosome status(Ph−ve vs Ph + ve)	0.877(0.400–1.922)	0.743
Diagnosis to transplant lag	1.000(0.985–1.014)	0.946

*HR* hazard ratio, *CI* confidence interval

**Table 5:** Analysis of risk factors of relapse among study participants

Variables	Relapsed		Non relapsed		OR	95% CI
	*n*	%	*n*	%		
Treatment group						
TBI/Cy	9	11.5	69	88.5	0.356	0.134–0.948
Bu/Cy	11	26.8	30	73.2		
Donor/recipient sex matching						
Mismatch	15	20.5	58	79.5	2.121	0.714–6.297
Matching	5	10.9	41	89.1		
Age in years						
< 30	12	16.4	61	83.6	0.934	0.350–2.495
> 30	8	17.4	38	82.6		
Phenotype						
B	11	13.9	68	86.1	0.557	0.210–1.482
T	9	22.5	31	77.5		
Philadelphia chromosome status						
Negative	15	16.3	77	83.7	0.857	0.280–2.621
Positive	5	18.5	22	81.5		
Disease status at transplant						
CR 1	6	10.7	50	89.3	0.420	0.149–1.182
≥ CR 2	14	22.2	49	77.8		
Recipient sex						
Females	12	14.0	74	86.0	0.507	0.186–1.382
Males	8	24.2	25	75.8		

*OR* Odds ratio, *CI* Confidence interval

### Conditioning regimen related toxicity

Severe mucositis was significantly higher in the TBI/Cy group (37.2% versus 9.8% in the Bu/Cy group). A significantly higher incidence of bacterial infections was found in the TBI group (43.6 versus 24.5 % in the Bu/Cy group). Both groups had a similar incidence of VOD and hemorrhagic complications. Although eight patients (17.8%) had CMV reactivation after the transplant, this could not be proven when tested by multivariate analysis. In the TBI/Cy group, three patients developed idiopathic pneumonitis, and five patients developed osteoporosis as a complication of TBI regimen related toxicity (Table 6).

**Table 6:** Comparison between the studied groups according to regimen-related toxicity

Variable	TBI/Cy (*n* = 78)	Bu/Cy (*n* = 41)	*p* value
**Mucositis**			0.005
Negative	27 (34.6)	16 (39.0)	
Mild	12 (15.4)	8 (19.5)	
Moderate	10 (12.8)	13 (31.7)	
Severe	29 (37.2)	4 (9.8)	
**Veno-occlusive disease**			0.691
Negative	74 (94.9)	38 (92.7)	
Positive	4 (5.1)	3 (7.3)	
**Hemorrhagic cystitis**			0.100
Negative	71 (91.0)	33 (80.5)	
Positive	7 (9.0)	8 (19.25)	
**Idiopathic pneumonitis**			0.278
Negative	75 (96.2)	41 (100.0)	
Positive	3 (3.8)	0 (0.0)	
**Diffuse alveolar**			0.545
Negative	76 (97.4)	41 (100.0)	
Positive	2 (2.6)	0 (0.0)	
**Osteoporosis**			0.116
Negative	73 (93.6)	41 (100.0)	
Positive	5 (6.4)	0 (0.0)	
**Infections**			0.037
Negative	40 (51.2)	29 (70.7)	
Bacterial	34 (43.6)	10 (24.5)	
Fungal	2 (2.6)	1 (2.4)	
Viral	2 (2.6)	1 (2.4)	
**CMV reactivation**			0.017
Negative	73 (93.6)	32 (78.0)	
Positive	5 (6.4)	9 (22.0)	

Variables are expressed as number and %

## Discussion

TBI-based regimens are widely used for ALL patients and are showing excellent outcomes without increasing the relapse rate. However, to avoid the wide range of long and short-term complications of TBI, the alternative radiation-free regimens based on Bu were introduced with the ability to produce comparable clinical results with lower transplant-related mortality and morbidity [3]. In the present study, we compared the clinical outcomes in two groups of ALL patients who underwent allo-HSCT using TBI/Cy conditioning versus a radiation-free regimen of oral Bu plus Cy.

Similar OS, DFS, and NRM were observed in both treatment groups; using multivariate analysis, the conditioning regimen was not an independent risk of OS, DFS, or NRM. However, patients who received a transplant in ≥ CR2 had lower DFS in both groups. Relapse remains the leading reason for treatment failure, and a common cause of death in ALL patients received allo-HSCT. Using TBI is associated with lower relapse rates in many patients [11]. In this study, using the Bu/Cy regimen was accompanied by a significantly higher relapse rate that was confirmed in the multivariate analysis.

Recent research by Wang and colleagues who carried a retrospective analysis on 224 adult patients with ALL in Taiwan noted similar results; patients who received Bu/Cy or TBI conditioning had similar OS, DFS, and NRM. Disease status before HSCT was the only risk factor of survival in his patients with poor DFS in patients transplanted in ≥ CR2 [12]. Another recent study published by the Center of International Blood and Marrow Transplantation (CIBMTR) concluded that the Bu/Cy patients had significantly more relapses than the TBI patients (HR, 1.46, 95% CI 1.15 to 1.85, *p* = .002) [13], which was similar to our results. The advantage of TBI over busulfan in reducing post-transplant relapse was also confirmed in a large analysis by the European Society for Blood and Marrow Transplantation (EBMT) that reported a high rate of relapse in the Bu/Cy group of ALL patients transplanted in CR1 [14].

Unlike our findings, the authors of a recent meta-analysis including over 800 ALL patients reported lower DFS, better NRM with the oral Bu/Cy regimens than the TBI ones, and a similar risk of relapse [15]. In the Japanese retrospective analysis by Mitsuhashi and colleagues [16], in addition to a large meta-analysis of fifteen non-randomized comparative studies of 6280 patients [17], a lower NRM with TBI compared to oral Bu/Cy conditioning was reported without a significant difference in the relapse risk between both regimens, which was against our findings.

A similar incidence of acute and chronic GVHD was observed among patients in both groups of the study, and this was similar to the findings published by Eroglu et al. [18].

For most MAC regimens including TBI, the incidence of severe mucositis ranged from 30 to 70%, increasing the risk of febrile neutropenia, serious infections, and the need for parenteral nutrition or narcotic therapy for pain control [19]. In this study, we demonstrated a significantly higher incidence of severe mucositis and bacterial infections associated with the use of TBI.

Previous studies reported an increase in the risk of VOD with the oral busulfan due to unpredictable absorption, supporting the use of the intravenous form to decrease the risk of such a fatal complication [5, 20]. Although fatal VOD was present in three patients in the Bu/Cy group compared to one patient in the TBI/Cy group, we could not find a significant impact of the conditioning regimen on the VOD risk in the multivariate analysis. Similarly, Sakellari et al. [21] found no significant difference in the VOD incidence among ALL patients who received TBI or Bu-based conditioning regimens. In contrast, we disagreed with the findings of a retrospective study by Kalaycio et al. who noted a higher incidence of VOD in patients received oral busulfan compared to those in the TBI group [22].

It should be noted that our study did not provide data on the plasma level of oral busulfan due to it was unavailable in a standardized lab in our country during the study period; it is unknown to what degree the dose adjustment according to the oral busulfan plasma level may have contributed to our findings.

Among our study participants, sepsis was the main cause of death in the TBI/Cy group, followed by relapse and organ failure. While in the Bu/Cy group, relapse was the leading cause of mortality followed by respiratory failure and sepsis. These findings were similar to a recent report from the CIBMTR [23].

## Conclusion

The two regimens offer sufficient immunosuppression facilitating engraftment, without significant difference in OS or DFS. The Bu/Cy regimen was associated with a higher risk of relapse compared to the TBI-based one. In this study, the TBI-based regimen appears to be slightly superior to the Bu/Cy one in terms of low relapse rates. The variability in the absorption and metabolism of the oral busulfan formulation could be the cause of plasma-level differences that can cause many conflicting results and toxicity with the two regimens.

## Study limitations

We must address the limitations of this study. This is a retrospective study with a relatively small number of participants. It is also worth mentioning that the intravenous Bu could not be used due to unavailability in our country. Despite these limitations, we had a relatively homogenous group of patients with similar baseline characteristics who received transplants in a large transplantation center using the standard procedures and had the advantage of a long period of follow-up in most of the patients. Our data might provide the basis for further larger prospective studies aiming at optimizing the conditioning regimens in adult ALL.

## Data Availability

The datasets used and/or analyzed during the current study are available from the corresponding author on reasonable request. The baseline data that support the finding of this work were obtained from BMT unit registry of Nasser institute after permission.

## Abbreviations

Allo-HSCT: Allogeneic hematopoietic stem cell transplantation
ALL: Acute lymphoblastic leukemia
CIBMTR: Center of international blood and marrow transplantation registry
DFS: Disease-free survival
EBMT: The European Society for Blood and Marrow Transplantation
GVHD: Graft versus host disease
G-CSF: Granulocyte colony-stimulating growth factors
MAC: Mayloablative conditioning
NRM: Non-relapse mortality
OS: Overall survival
VOD: Veno-occlusive disease
